# Predictors of Mortality Among Hemodialysis Patients at Al-Thora General Hospital, Ibb Governate, Yemen: A Retrospective Study

**DOI:** 10.7759/cureus.65457

**Published:** 2024-07-26

**Authors:** Abdulghani Ghabisha, Ismaeel A AlShoaibi, Faisal Ahmed, Saif A Ghabisha, Basheer Abdo

**Affiliations:** 1 Department of Internal Medicine, School of Medicine, Ibb University, Ibb, YEM; 2 Department of Urology, Ibb University, Ibb, YEM; 3 Department of General Surgery, Ibb University, Ibb, YEM

**Keywords:** yemen, ibb, mortality, survival, predictive factor, hemodialysis, end-stage renal disease

## Abstract

Background: In addition to the global rise in the use of hemodialysis (HD) for end-stage renal disease, individuals receiving maintenance HD continue to have higher mortality rates than the general population. The mortality rates among HD patients in Yemen have not been studied because of the lack of a national registry system, and the impact of the disease on the country is yet to be evaluated. Our study aimed to assess the clinical characteristics and factors associated with mortality among patients with HD in a resource-limited setting.

Materials and methods: This retrospective study involved 4194 HD patients at the Nephrology Center of Al-Thora General Hospital, Ibb Governate, Yemen, between March 2014 and September 2023. Data on HD patients' demographic characteristics, risk factors, and comorbidities were gathered and analyzed. The Kaplan-Meier and log-rank tests were used to evaluate and compare survival curves, and the proportional Cox hazard model was used to investigate the factors associated with mortality.

Result: The mean age was 49.2 ± 16.5 years. The majority of cases were male (n= 2604, 62.1%) and from rural areas (3386, 80.7%), with 1226 (29.2%) living outside Ibb Governorate. Hepatitis C and B viruses were positive in 466 (11.1%) and 312 (7.4%) patients. The main comorbidity was hypertension (n= 3152, 75.2%), followed by diabetes mellitus (DM) (n= 1375, 32.8%). Five hundred and forty-eight patients died during the study period between 2017 and 2023, with an estimated mortality rate of 13.1%. The survival rates at 12, 24, 36, 48, and 60 months of follow-up were approximately 97.4%, 93.3%, 91.7%, 86.0%, and 74.6%, respectively. Predictive factors for mortality among HD patients in the Cox regression model were age >65 years (HR:1.41; 95 % CI: 1.15-1.74, p<0.001), cardiovascular disease (HR: 7.28; 95 % CI: 2.68-19.81, p<0.001), coming from other cities (HR: 1.32; 95% CI: 1.11-1.59, p= 0.002), DM (HR: 1.58; 95% CI: 1.23-2.01, p <0.001), and cerebral vascular accidents (HR:1.57; 95 % CI: 1.13-2.18, p= 0.007).

Conclusion: Instead of a higher mortality rate in this study, coming from other cities, DM, cardiovascular disease, cerebral vascular accidents, and age >65 years were predictive factors for mortality in HD patients. The study underlines the necessity of planning new HD facilities, avoiding and treating comorbidities, managing them early to decrease mortality, and educating regional administrative decision-makers on effective implementation techniques.

## Introduction

Recently, the incidence of renal failure and chronic kidney disease (CKD) has increased and become a major global health problem [1]. CKD is a significant death risk factor, ranking 13th globally in 2016, and is projected to become the fifth leading cause of death by 2040 [2]. The need for hemodialysis (HD) represents 10% of patients with CKD and is continuously increasing. Despite advances in HD technology, individuals undergoing HD have a 10- to 30-fold higher mortality rate than the general population [3]. The prevalence of end-stage kidney disease (ESKD) in the Middle East is 55-818 per million people [4]. Identifying risk factors and therapeutic targets is critical for improving individual and community health [1]. Impaired fasting plasma glucose levels, hypertension, sodium-rich diet, high body mass index (BMI), and lead exposure are common risk factors for CKD development [4,5]. Furthermore, hypertension is the primary cause of CKD in Eastern Europe, East Asia, tropical Latin America, and Western Sub-Saharan Africa. In contrast, high fasting plasma glucose is the primary risk factor in all other locations [6].

The prevalence of CKD in developing countries is unclear because of a lack of national registries and community-based investigations [7]. Furthermore, dialysis results in these nations are highly dismal, with survival rates ranging from 20 to 70% [8]. According to the International Committee of the Red Cross, four of Yemen's 32 dialysis centers have closed since the conflict, with the remaining 28 failing to provide services owing to broken equipment, a lack of critical supplies, and underpaid workers [9]. In Yemen, the perilous situation forced patients to limit their visits to two dialysis per week. Since the conflict began in 2015, 25% of Yemen's dialysis patients have died every year due to a lack of dialysis supplies, working dialysis equipment, and staff compensation [9,10].

Like other developing countries, Yemen did not have precise national registries and community-based investigations regarding CKD. Additionally, the mortality rates among HD patients in Yemen have not been studied, and the impact of the disease on the country is yet to be evaluated. Given the scarcity of data on HD patients in Yemen, this study aimed to assess the clinical characteristics and factors associated with mortality among HD patients in a resource-limited setting.

## Materials and methods

Study design and population

This was a retrospective study involving 4194 HD patients in the Nephrology Center of Al-Thora General Hospital, Ibb Governate, Yemen, between March 2014 and September 2023, for eight years. The primary outcome was death, and all patients were monitored until death or censoring (end of follow-up, loss to follow-up, transfer, or kidney transplant). Data were gathered from patients' medical records and a computerized record system at our Al-Thora General Hospital, Ibb Governorate, Yemen facility. The hemodialysis center located at Al-Thora General Hospital is the only one in our city and serves HD patients in the Ibb Governorate. Furthermore, since 2015, it has covered additional HD patients, including the Taiz Governate and other governments that stopped or did not operate owing to restrictive policies during the present conflicts [9,10]. Patients on chronic dialysis for less than three months, those with cancer at the time of inclusion, and pregnant women were excluded.

Collected data

Demographic characteristics such as age, age classes (< 65, ≥ 65 years), gender (male and female), residency (rural or urban), city (Ibb Governate and other cities), comorbidities such as hypertension, thyroid disease, diabetes mellitus (DM), cardiovascular disease, cerebral vascular accidents, viral hepatitis infections with viruses C and B, chronic liver disease, and outcome (live or death) were collected from the patient's medical records. Overall survival (OS) and mortality were calculated from the time of starting HD to death, switched over to other forms of renal replacement therapy like continuous ambulatory peritoneal dialysis and renal transplantation, or the endpoint of follow-up. Causes of death were identified by differentiating deaths directly related to CKD from those caused by other causes. The primary outcome measure was mortality. The secondary outcome was the predictive factor for mortality in HD patients.

Statistical analysis

We conducted a descriptive analysis of the entire sample, reporting quantitative data as mean ± standard deviation and qualitative variables as frequencies and percentages. To verify the normal distribution of the study's variables, we utilized the Kolmogorov-Smirnov test. To discern significant associations between qualitative variables, we implemented the chi-square test. In instances where the expected frequency was restricted, Fisher's exact test was deemed appropriate. The Kaplan-Meier estimator initially characterized the distribution of time to death. All variables were analyzed univariately (log-rank test). To assess the influence of several predictors of mortality from any cause, we utilized the Cox proportional hazards model, which included the factors most strongly linked with survival. A univariate Cox model was used to investigate the relationship between each explanatory variable and the time to death. Variables with a p-value less than 0.20 in univariate analysis were added to the multivariate Cox model. For all analyses, the significance level was set at p < 0.05. All analyses were done using IBM SPSS Statistics for Windows, Version 23 (Released 2015; IBM Corp., Armonk, New York, United States).

Ethical approval

The study was conducted in accordance with the Declaration of Helsinki and was approved by the Ibb University Institutional Ethics Committee (Code: IBBUNI. AC. YEM. 2024.72 on 2024-02-03). Owing to the study's retrospective nature, written informed consent from the included patients was not required.

## Results

The mean age was 49.2 ± 16.5 years. Most cases were male (n= 2604, 62.1%), from rural areas (n= 3386, 80.7%), and out of Ibb Governate (n= 1226, 29.2%). Hepatitis C virus and Hepatitis B virus were positive in 466 (11.1%) and 312 (7.4%) of patients, respectively. The main comorbidities were hypertension (n= 3152, 75.2%) and DM (n= 1375, 32.8%) (Table 1).

**Table 1:** Clinical and biological characteristics of the study population. SD: Standard deviation

Variables	N (%)
Age (year), Mean ± SD	49.2 ± 16.5 (range: 8.0 - 100.0)
Age groups	
≤ 65 years	3526 (84.1%)
> 65 years	668 (15.9%)
Gender	
Male	2604 (62.1%)
Female	1590 (37.9%)
Residency	
Urban	808 (19.3%)
Rural	3386 (80.7%)
City	
Ibb	2331 (55.6%)
Taiz	637 (15.2%)
Others	1226 (29.2%)
Laboratory viral result	
Hepatitis C virus	466 (11.1%)
Hepatitis B virus	312 (7.4%)
Comorbidities	
Hypertension	3152 (75.2%)
Diabetic mellitus	1375 (32.8%)
Chronic liver disease	367 (8.8%)
Thyroid disease	356 (8.5%)
Cerebral vascular accident	179 (4.3%)
Cardiovascular disease	175 (4.2%)

**Figure 1:**
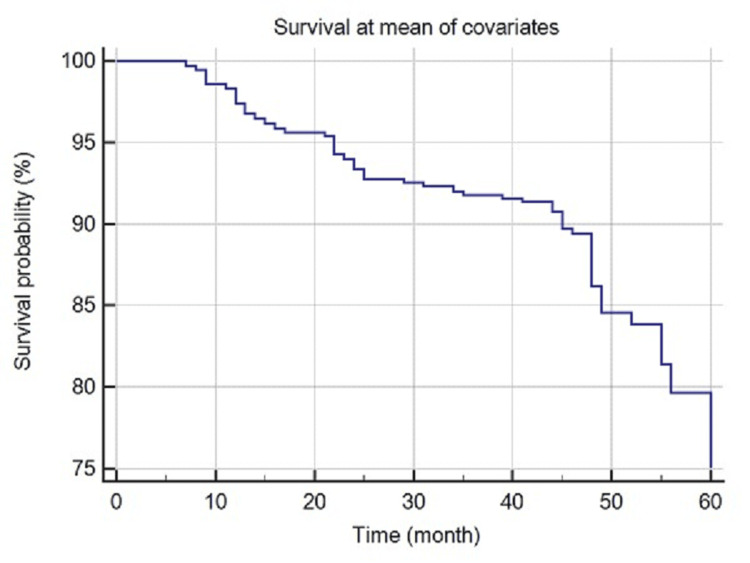
Kaplan–Meier survival curves for overall survival in hemodialysis cases.

Five hundred and forty-eight patients died during the study period between 2017 and 2023, with an estimated mortality rate of 13.1%. The overall survival at 12 months of follow-up was approximately 97.4% [95% Confidence Intervals (CI): 96.9%-97.9%], 93.3% (95% CI: 92.5%-94.0%) at 24 months, 91.7% (95% CI: 90.9%-92.5%) at 36 months, 86.0% (95% CI: 84.8%-87.2%) at 48 months, and 74.6% (95% CI: 71.4%-77.9%) at 60 months, as shown in Figure 1.

The analysis of the results obtained showed that the surviving patients were younger than the deceased patients (48.9 ±16.5 years vs. 51.2 ±16.3 years old, p=0.002). Additionally, living in rural areas (p<0.001) and viral infections with HCV and HBV were less common among surviving patients and were statistically significant (p<0.001 and 0.016; respectively). Furthermore, comorbidities like hypertension (p<0.001), diabetic mellitus (p<0.001), cardiovascular disease (p<0.001), cerebrovascular disease (p<0.001), and chronic liver disease (p<0.001) were less common among surviving patients and were statistically significant (Table 2).

**Table 2:** Comparison between survivors and decades of hemodialysis patients.

Variables	Subgroups	Survivals (n=3646)	Decades (n=548)	p-value
Age (year)	Mean ± SD	48.9 ±16.5	51.2±16.3	0.002
Gender	Male	2260 (62.0)	344 (62.8)	0.759
	Female	1386 (38.0)	204 (37.2)	
Cities	Ibb city	2010 (55.1)	321 (58.6)	0.142
	Others cities	1636 (44.9)	227 (41.4)	
Residency	Urban	665 (18.2)	143 (26.1)	<0.001
	Rural	2981 (81.8)	405 (73.9)	
Hepatitis C virus	No	3280 (90.0)	448 (81.8)	<0.001
	Yes	366 (10.0)	100 (18.2)	
Hepatitis B virus	No	3389 (93.0)	493 (90.0)	0.016
	Yes	257 (7.0)	55 (10.0)	
History of hypertension	No	844 (23.1)	198 (36.1)	<0.001
	Yes	2802 (76.9)	350 (63.9)	
History of diabetic mellitus	No	3146 (86.3)	405 (73.9)	<0.001
	Yes	500 (13.7)	143 (26.1)	
History of cerebrovascular diseases	No	3565 (97.8)	450 (82.1)	<0.001
	Yes	81 (2.2)	98 (17.9)	
History of cardiovascular cerebrovascular disease	No	3557 (97.6)	462 (84.3)	<0.001
	Yes	89 (2.4)	86 (15.7)	
History of thyroid disease	No	3467 (95.1)	371 (67.7)	<0.001
	Yes	179 (4.9)	177 (32.3)	
History of liver disease	No	3461 (94.9)	366 (66.8)	<0.001
	Yes	185 (5.1)	182 (33.2)	

Predictive factors for mortality

**Figure 2:**
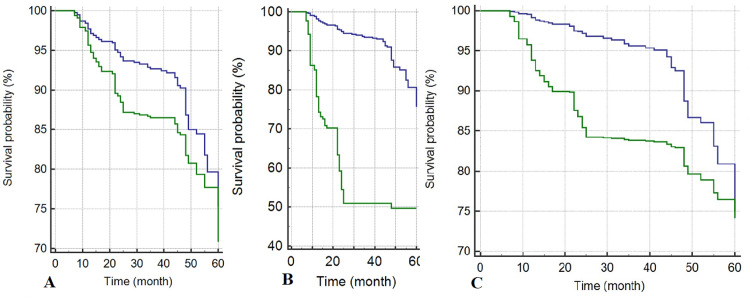
A: Survival plots divided by age (age> 65 years: Green line) (age≤ 65 years: Blue line). B: Survival plots divided by cardiovascular disease (present: Green line) (absent: Blue line). C: Survival plots divided by cerebral vascular accidents (present: Green line) (absent: Blue line).

**Figure 3:**
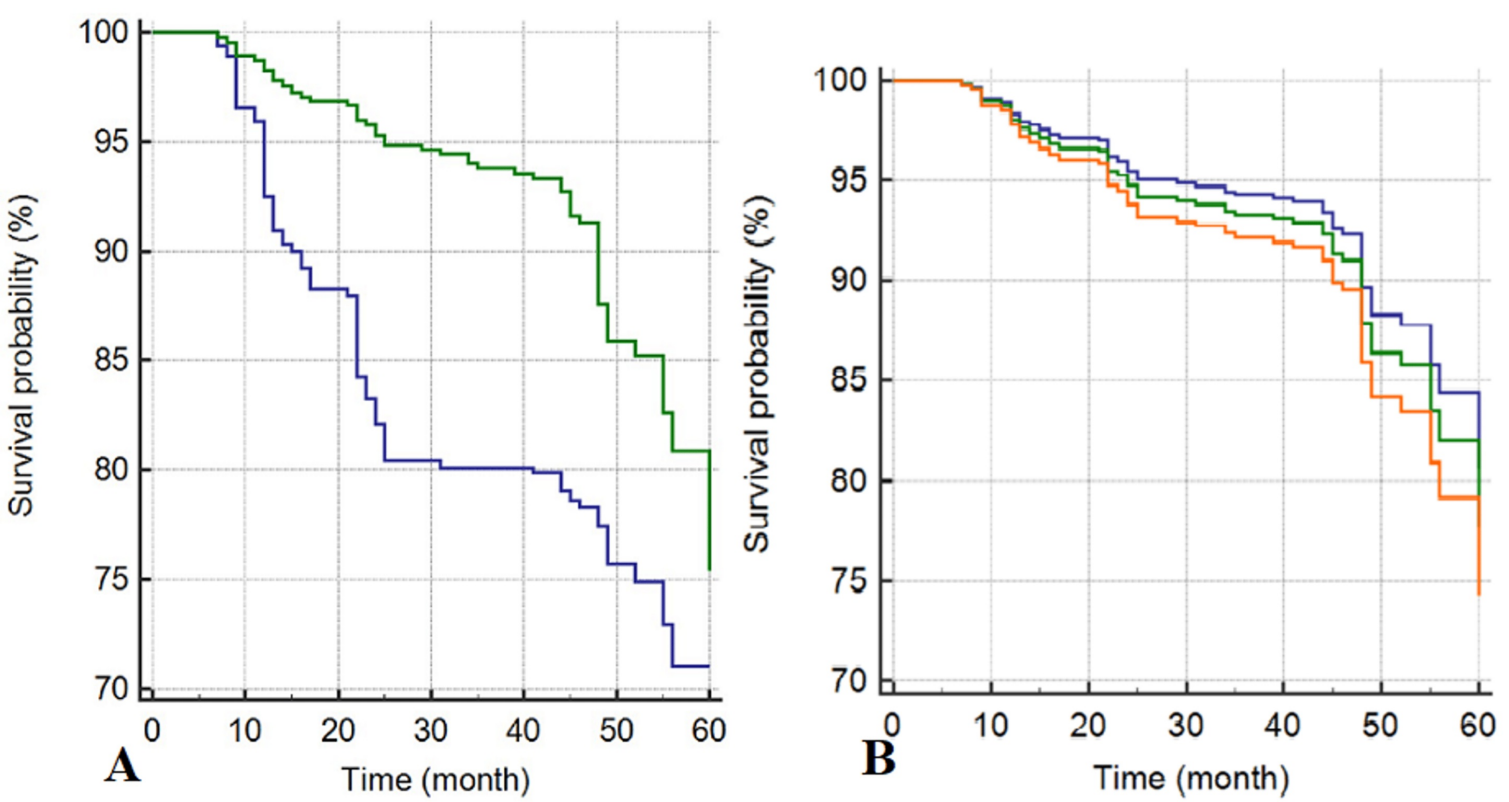
A: Survival plots divided by diabetes mellitus (present: Green line) (absent: Blue line). B: Survival plots divided by residency (other cities: Green line) (Ibb city: Blue line) (Taiz city: Orange line).

Predictive factors for mortality among HD patients in the Cox regression model were age > 65 years (HR:1.41; 95 % CI: 1.15-1.74, p<0.001) (Figure 2A), cardiovascular disease (HR: 7.28; 95 % CI: 2.68-19.81, p<0.001) (Figure 2B), cerebral vascular accidents (HR:1.57; 95 % CI: 1.13-2.18, p= 0.007) (Figure 2C) and DM (HR: 1.58; 95% CI: 1.23-2.01, p <0.001) (Figure 3A), and coming from other cities (HR: 1.32; 95% CI: 1.11-1.59, p= 0.002) (Figure 3B) (Table 3).

**Table 3:** Predictors of mortality according to the Cox proportional hazards model. SD: Standard deviation, HR: hazard ratio. CI: confidence interval.

Variables	Subgroup	Total	Univariate analysis		Multivariate analysis	
			HR (95% CI)	p-value	HR (95% CI)	p-value
Gender	Male	2598 (62.0)	Reference group	0.559	Reference group	0.788
	Female	1590 (38.0)	0.95 (0.80-1.13)		0.98 (0.82-1.16)	
Residency	Urban	806 (19.2)	Reference group	<0.001	Reference group	0.450
	Rural	3382 (80.8)	0.62 (0.51-0.75)		0.92 (0.75-1.14)	
City	Ibb	2331 (55.6)	Reference group		Reference group	0.002
	Other	1863 (44.4)	0.94 (0.79-1.11)	0.439	1.32 (1.11-1.59)	
Hepatitis C virus	No	3722 (88.9)	Reference group	<0.001	Reference group	0.740
	Yes	466 (11.1)	2.03 (1.63-2.52)		0.96 (0.75-1.23)	
Hepatitis B virus	No	3880 (92.6)	Reference group	0.003	Reference group	0.910
	Yes	308 (7.4)	1.52 (1.15-2.01)		1.02 (0.75-1.37)	
Hypertension	No	1041 (24.9)	Reference group	<0.001	Reference group	0.194
	Yes	3147 (75.1)	0.55 (0.46-0.65)		0.86 (0.69-1.08)	
Diabetic mellitus	No	2817 (67.3)	Reference group	<0.001	Reference group	<0.001
	Yes	1371 (32.7)	2.55 (2.16-3.02)		1.58 (1.23-2.01)	
Cerebral vascular accident	No	4009 (95.7)	Reference group	<0.001	Reference group	0.007
	Yes	179 (4.3)	7.15 (5.74-8.91)		1.57 (1.13-2.18)	
Cardiovascular disease	No	4013 (95.8)	Reference group	<0.001	Reference group	<0.001
	Yes	175 (4.2)	6.32 (5.01-7.95)		7.28 (2.68-19.81)	
Thyroid disorder	No	3832 (91.5)	Reference group	<0.001	Reference group	0.937
	Yes	356 (8.5)	8.02 (6.69-9.60)		1.03 (0.54-1.97)	
Chronic liver disease	No	3821 (91.2)	Reference group	<0.001	Reference group	0.600
	Yes	367 (8.8)	7.94 (6.64-9.49)		0.79 (0.33-1.89)	
Age (year)	≤ 65 years	3094 (84.9)	Reference group	<0.001	Reference group	0.0010
	> 65 years	552 (15.1)	1.48 (1.21-1.82)		1.41 (1.151 to 1.74)	

## Discussion

In this study, we investigated the prevalence of mortality and its predictive factors among HD patients during 10 years in the Ibb Governate, Yemen. The predictors of mortality were coming from other cities, DM, cardiovascular disease, cerebrovascular accidents, and age >65 years. Our study's mortality rate among the HD patients was 13.1%. Our result was higher than the previously reported mortality rates among HD patients from Qatar (6.4%) and Somalia (5.2%) [11,12]. However, this rate was mainly comparable to the crude mortality rate in Europe (15.6%) [13].

In agreement with the findings of other studies, our findings showed that DM was a predictive factor for mortality in HD patients (HR= 1.58) [12,14,15]. When ESRD patients with DM were compared to non-diabetic ESRD patients, the risk of death was considerably greater, demonstrating that DM carries an independent mortality risk that may be attributed to the buildup of end-organ damage and inflammatory and oxidative stress mediators [16,17]. We also found that age >65 years and cardiovascular disease were correlated with higher mortality in patients with HD. The hazard ratio of death was 1.41 and 7.28 for age >65 years and cardiovascular disease, respectively. Older age, cardiovascular disease, and their association with mortality in HD patients are well understood in the renal literature [18]. Additionally, these findings were similar to the recent findings of a systematic review and meta-analysis [19]. Aging gradually alters the structure and function of the vasculature, causing hemodynamic disturbances owing to increased oxidative stress, early cellular senescence, and impairments in the synthesis and/or release of endothelial-derived vasoactive molecules [20]. Wu's study on ESKD mortality in China from 1990-2019 revealed that the likelihood of ESKD deaths increases exponentially with age, possibly due to older patients' delicate physiological processes and immune systems [21].

Our results showed that a history of cerebrovascular disease was a predictive factor for mortality in HD patients (HR=1.57). This finding was similar to a recent systematic review and meta-analysis and study by Goodkin et al. [19,22]. However, hypertension was not a predictive factor of mortality in our study. A similar result was reported by Msaad et al., who found that hypertension was not a contributing factor to death [3]. Mortality risk factors vary by country and according to different reports. Differences in mortality risk exist between nations, HD centers, and geographic locations, determined mainly by the group of patients studied and the data gathered [23].

Overall, we found no significant sex differences affecting the outcomes, either mortality or survival, among the recruited HD patients. Because there were more male than female patients in the current study, the discrepancies in death rates may be influenced by the sex ratio; that is, 13.24% of men and 12.83% of females died, whereas 86.76% of males and 87.17% of females survived. Ejaz et al. reported similar findings [24]. In contrast, Chandrashekar et al. found that females were more likely to die than males [25].

In this study, moving from other cities predicted mortality among the patients undergoing HD. Patients from these cities find it difficult to get to the center because they are far away and expensive to travel, resulting in a longer HD interval, a higher frequency of uremic complications, higher socioeconomic costs, and, ultimately, increased death. These findings are similar to those of previous reports [24,26]. According to a 2011 Australian survey, most patients spend between 10 and 50 $ per week on transportation, with reduced travel and reduced family hardship being the most popular reasons for choosing home-based peritoneal dialysis versus HD [26]. In another report from the United States Renal Data System, HD withdrawal caused death in approximately 15% of total deaths [27]. Travel burden and equitable access to dialysis should be the top objectives when creating new dialysis infrastructure, and future research should look into patient-, service-, and state-level factors that determine whether a patient can receive dialysis near home [28].

Study limitations

The main limitation of this study is its reliance on secondary data, the quality of which can be inconsistent due to variations in documentation, data integrity, and record-keeping practices. Furthermore, the retrospective nature of this study may have introduced inherent biases. Excluding records with incomplete data could also introduce a selection bias in the analysis. Nonetheless, by providing data on possible predictive markers for death among HD patients, our findings contribute significantly to the literature on survival in maintenance HD patients. Another limitation is that the study did not look at other potential factors such as serum albumin, body mass index, malnutrition, or inflammatory markers that could have influenced the result of survival. For that, the results on the influence of baseline indicators on patient survival time, in particular, may not reflect those from other countries. Another limitation is that most of our patients were on twice-weekly HD that offered eight hours of dialysis per week, but guidelines recommend more frequent and longer duration for those with residual renal function > 2 ml/min. We could not adhere to the above guidelines due to overcrowding and financial constraints, and difficult to perform for cases outside of the Ibb city. We recommend conducting a prospective study with a larger sample size and an extended postoperative follow-up to mitigate these limitations and provide more robust findings.

## Conclusions

Instead of a higher mortality rate in this study, coming from other cities, DM, cardiovascular disease, cerebral vascular accident, and age >65 years were predictive factors for mortality in HD patients. The study underlines the need to plan for new HD facilities, avoid and treat comorbidities, manage them early to decrease morbidity and mortality, and educate regional administrative decision-makers on effective implementation techniques. Future research including controlled studies employing risk prediction models to assess the efficacy of preventative interventions and a larger sample size that includes patient data from several hospitals for external validation is recommended.
